# Physical Perfection

**Published:** 1893-01

**Authors:** 


### PHYSICAL PERFECTION.

The selection of a model for the Silver Statue which is to crown Montana's World's Fair Edifice, has given rise to some measure of jealousy among some of the more prominent female members of the histrionic art, who, for some reason, deem *theirs* the exclusive field from which the selection should quite naturally have been made, perhaps because the play going public has become more familiar with unadorned female loveliness in that quarter than in any other.

Some few years ago, Harvard University offered a prize to the man or woman who should show the nearest approach to physical perfection in proportion. The young woman who gained the prize was 25 years of age, 5 feet 5 inches in height, and weighed 130 lbs. The male winner of the prize was 6 feet 1 inch in height. Dr. Sargent gives the following table of average heights, which he prepared from the statistics of Dr. Weisbach, of Constantinople, who has measured hundreds of individuals of many different races.

| | Feet. | Inches. |
|---|---|---|
| Henry C. Jackson, Maine | 6 | 1 |
| New Zealand Maoris | 5 | 10⅙ |
| Kafirs of Africa | 5 | 10 |
| Norwegians, average | 5 | 9 |
| Scotch, average | 5 | 8⅓ |
| Swedes, average | 5 | 8 |
| English and Irish, average | 5 | 7⅔ |
| Danes, average | 5 | 7½ |
| Germans, average | 5 | 7¼ |
| Italians, average | 5 | 6¼ |
| French, average | 5 | 6¼ |
| Spanish and Portuguese, average | 5 | 6 |
| Jews, average | 5 | 3 |

